# Angiotensin II Moderately Decreases *Plasmodium* Infection and Experimental Cerebral Malaria in Mice

**DOI:** 10.1371/journal.pone.0138191

**Published:** 2015-09-16

**Authors:** Julio Gallego-Delgado, Charlotte Baravian, Innocent Edagha, Maureen C. Ty, Marta Ruiz-Ortega, Wenyue Xu, Ana Rodriguez

**Affiliations:** 1 New York University School of Medicine, Dept. of Microbiology, Division of Parasitology, New York, New York, United States of America; 2 Cellular Biology in Renal Diseases Laboratory, IIS-Fundación Jiménez Díaz/Universidad Autónoma Madrid, Madrid, Spain; 3 Department of Pathogenic Biology, Third Military Medical University, Chongqing, China; Université Pierre et Marie Curie, FRANCE

## Abstract

Angiotensin II, a peptide hormone that regulates blood pressure, has been proposed as a protective factor against cerebral malaria based on a genetic analysis. *In vitro* studies have documented an inhibitory effect of angiotensin II on Plasmodium growth, while studies using chemical inhibitors of angiotensin II in mice showed protection against experimental cerebral malaria but not major effects on parasite growth. To determine whether the level of angiotensin II affects Plasmodium growth and/or disease outcome in malaria, elevated levels of angiotensin II were induced in mice by intradermal implantation of osmotic mini-pumps providing constant release of this hormone. Mice were then infected with *P*. *berghei* and monitored for parasitemia and incidence of cerebral malaria. Mice infused with angiotensin II showed decreased parasitemia seven days after infection. The development of experimental cerebral malaria was delayed and a moderate increase in survival was observed in mice with elevated angiotensin II, as confirmed by decreased number of cerebral hemorrhages compared to controls. The results presented here show for the first time the effect of elevated levels of angiotensin II in an *in vivo* model of malaria. The decreased pathogenesis observed in mice complements a previous human genetic study, reinforcing the hypothesis of a beneficial effect of angiotensin II in malaria.

## Introduction

Malaria is still a major public health problem world wide, causing more than 200 million cases per year and approximately 600,000 deaths, mostly in African children [1]. Of those that die from malaria, a high proportion succumb to cerebral malaria, a syndrome characterized by impaired consciousness, generalized convulsions, coma and neurological sequelae [2]. The interaction between *Plasmodium falciparum* infected red blood cells and host endothelial cells plays a key role in cerebral malaria. Mature stage parasites express ligands (PfEMP1) on the surface of infected erythrocytes that interact with host endothelial cell receptors (Protein C receptor, ICAM-1 [3, 4]) leading to their sequestration into the brain microcirculation, promoting the loss of endothelial cell junctions, endothelial apoptosis, and ultimately the disruption of the blood-brain barrier. This disruption causes a massive diffusion of blood cells and serum into the brain tissue leading to coma and damage to the nervous system [5].

Angiotensin II (Ang II) is a peptide hormone with well-characterized effects on circulatory homeostasis, where it induces vasoconstriction that results in increased high blood pressure. Ang II is derived from angiotensinogen through sequential enzymatic cleavages: first renin cleaves angiotensinogen, forming Ang I that is then converted to Ang II by angiotensin converting enzyme (ACE). Circulating Ang II not only contributes to increase blood pressure, but it is also involved in key inflammatory events, including the activation of endothelial cells to express higher levels of leukocyte adhesive molecules and the increase in vascular permeability [6].

A protective role for Ang II against cerebral malaria was proposed based on a gene polymorphism analysis of angiotensin-related enzymes in patients with severe or mild malaria, suggesting that elevated levels of Ang II would reduce the incidence of severe disease [7]. Additionally, Ang II was found to inhibit the growth of *P*. *falciparum in vitro* [8].

To study the effect of Ang II in malaria *in vivo*, an experimental model in mice exposed to increased levels of circulating Ang II was developed. Since Ang II has a rapid turnover [9], continuous delivery is required to maintain elevated levels in plasma. Using subcutaneous mini-pumps for constant delivery of Ang II in a malaria rodent model, it was observed that increased levels of Ang II result in a moderate decrease of levels of blood parasitemia and incidence of experimental cerebral malaria.

## Material and Methods

This study was carried out in strict accordance with the recommendations in the Guide for the Care and Use of Laboratory Animals of the National Institutes of Health. The protocol (IACUC#110910) was approved by the Institutional Animal Care and Use Committee of New York University School of Medicine, which is fully accredited by the Association For Assessment and Accreditation Of Laboratory Animal Care International (AAALAC).

### *P*. *falciparum* growth inhibition assay

*P*. *falciparum* 3D7 erythrocytic asexual cultures were maintained at 5% hematocrit in complete media (RPMI 1640, 25 mM HEPES, 10 μg/ml gentamycin, 0.5 mM hypoxanthine, pH 6.75), supplemented with 25 mM sodium bicarbonate and 0.5% Albumax II at 5% oxygen, 5% carbon dioxide and 90% nitrogen. Parasite cultures were synchronized using magnetic separation of schizont stages with MACS cell separation column (Miltenyi Biotec). *P*. *falciparum* infected erythrocytes in late stages were added to 96-well plates at 1.9% parasitemia 5% hematocrite and incubated for 24 h in the presence of different concentrations of Ang II (Bachem Americas, Inc., CA, USA). 10 μl from each well were smeared on glass slides and stained with Giemsa before blind microscopic quantification of parasites. To asses whether Ang II interferes in the process of erythrocyte rupture after the completion of the infection cycle, 5x10^5^ schizont stages per well (96% purity) were added to 96-well plates in the presence of different concentrations of Ang II (n = 8/dose) and incubated for 16 h and therefore analyzed independently to quantify the remaining non-ruptured erythrocytes in a standard hemocytometer.

### Mouse infection with *P*. *berghei*-ANKA and determination of experimental cerebral malaria

Mice 5–6 weeks old C57BL/6 were purchased from Taconic and infected with *Plasmodium berghei*-ANKA 10^6^ infected erythrocytes in 200 μl of sterile PBS i.p. Determination and severity of experimental cerebral malaria was scored based on appearance (Normal = 0; Coat ruffled = 1; Coat staring/panting = 2) and behavior (Normal = 0; Hunched = 1; Partial paralysis = 2; Convulsions = 3). This scoring system is a modification of a method described before [10]. Mice with score 2 or higher were considered positive for experimental cerebral malaria. Mice with a cumulative score of 3 or above were euthanized by CO2 narcosis followed by cervical dislocation. This occurred from day 5 to day 10 after infection, depending on the individual animals and groups. Mice that did not develop cerebral malaria were euthanized on day 12. Immediately after euthanasia, brains were collected and washed in cold PBS to remove excess of blood and fixed with paraformaldehyde 4% for up to 24 h and subsequent transferred to ethanol 70%.

### Implantation of subcutaneous osmotic mini-pumps

Mice were systemically infused with saline buffer (0.25 μl/h) or Ang II (100 or 500 ng/kg/min at 0.25 μl/h) (Bachem Americas, Inc., CA, USA) using osmotic mini-pumps that provide sustained 14-day delivery (Model 1002, DURECT Corp., Cupertino, CA, USA). Mini-pumps were implanted subcutaneously on the animal back right before infection.

### Brain histological analysis

Paraffin-embedded brain sections were stained with H&E and scanned on the Leica Biosystems SCN400 whole slide scanner. Experimental cerebral malaria induced histopathological alterations in the brain were assessed by counting and measuring the number and maximal diameter of petechiae and hemorrhages in an area of 41.6 ± 4.9 mm^2^ of brain histological sections per mouse using the Slidepath Digital Image Hub (DIH) software.

### Statistical analysis

**Fig 1:**
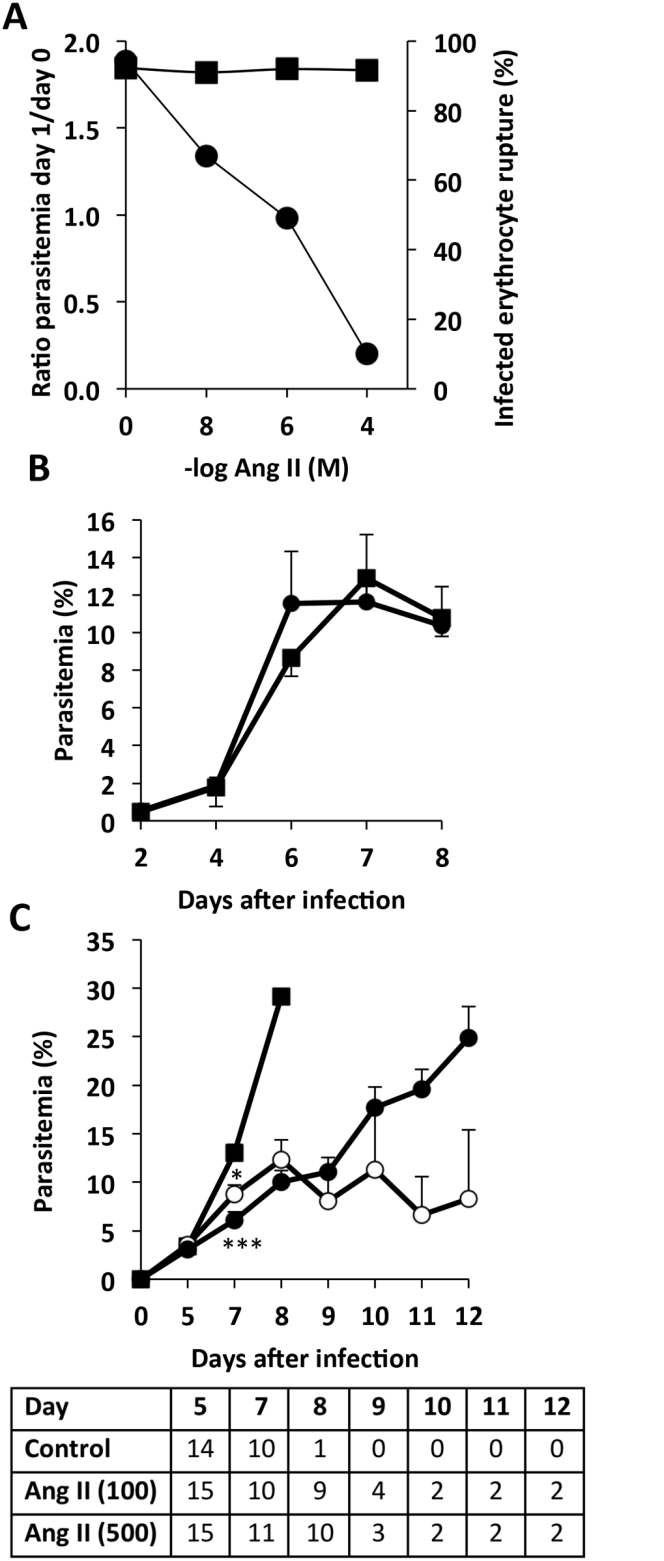
Ang II inhibits *P*. *falciparum* growth *in vitro* and *P*. *berghei* growth in mice. A) Parasite levels in cultures of *P*. *falciparum* incubated with different concentrations of Ang II *in vitro*. Ratio of day 0 (initial parasitemia) to day 1 (right axis, circles) and percentage rupture of infected erythrocytes (left axis, squares). B) Parasitemia was determined in groups of mice that were infected with *P*. *berghei* (day 0) and had been subjected (n = 8; circles) or not (n = 8; squares) to implantation of subcutaneous mini-pumps releasing saline buffer. C) Parasitemia of groups of mice infected with *P*. *berghei*: control (n = 14; circles), implanted with intradermal micro-pumps releasing Ang II at 100 ng/kg/min (n = 15; squares) or 500 ng/kg/min (n = 15; triangles). Number of surviving mice in each group are indicated in the table. Mice numbers decrease due to experimental cerebral malaria induced death (see Fig 2B). (B,C) Statistical analysis by t-Student (B) and Kruskal Wallis (C). Average plus standard error is shown. * *p*<0.05; **p<0.01; ***p<0.001.

Data on every experiment was analyzed to determine if it follows a normal distribution and if variance between groups is not different using Shapiro-Wilk and Levene’s tests for normality and homoscedasticity, respectively. Parasitemia levels in Fig 1C did not pass the tests and were analyzed using the Kruskal Wallis test on specific days. Data from every other experiment passed the tests for normality and was analyzed by t-Student or ANOVA. Cerebral malaria and survival were analyzed using Kaplan-Meier to determine differences among the groups. Pairwise log rank comparison was performed after applying Bonferroni correction (statistical significance accepted at the p <0.0167 level).

## Results and Discussion

To first characterize the anti-Plasmodium effect of Ang II, the activity of Ang II was tested against *P*. *falciparum* cultures *in vitro*. A dose-dependent inhibition of growth was observed, confirming previous reports in *P*. *gallinaceum* [11] and *P*. *falciparum* [8]. It was also observed that the activity does not plateau at higher concentrations of Ang II, but it reaches almost complete inhibition of infection at high (non-physiological) concentrations (Fig 1A).

To determine whether Ang II affects Plasmodium growth and disease outcome *in vivo*, the *P*. *berghei*-ANKA infection of C57BL/6 mice model of malaria was used. This combination of mouse strain and parasite species induces experimental cerebral malaria, a syndrome that mimics the pathogenesis of human cerebral malaria, despite differences in the initial mechanism of erythrocyte sequestration in the brain [12].

Since Ang II is rapidly degraded in plasma [13], to experimentally modify the levels of Ang II in mice, it is required to implant intra-dermal micro-pumps that provide a constant delivery of Ang II into the blood. We first confirmed that the implant of these pumps does not affect the development of *P*. *berghei* in mice (Fig 1B).

To study the role of Ang II in mice, we used two different concentrations of Ang II (100 and 500 ng/kg/min) previously shown to have no detectable effects in mouse blood pressure or other apparent pathology [14, 15]. When mice were treated with Ang II, we observed lower levels of parasitemia in these mice, compared to control infected mice (Fig 1C). This effect was only detectable after day 5 of infection.

**Fig 2:**
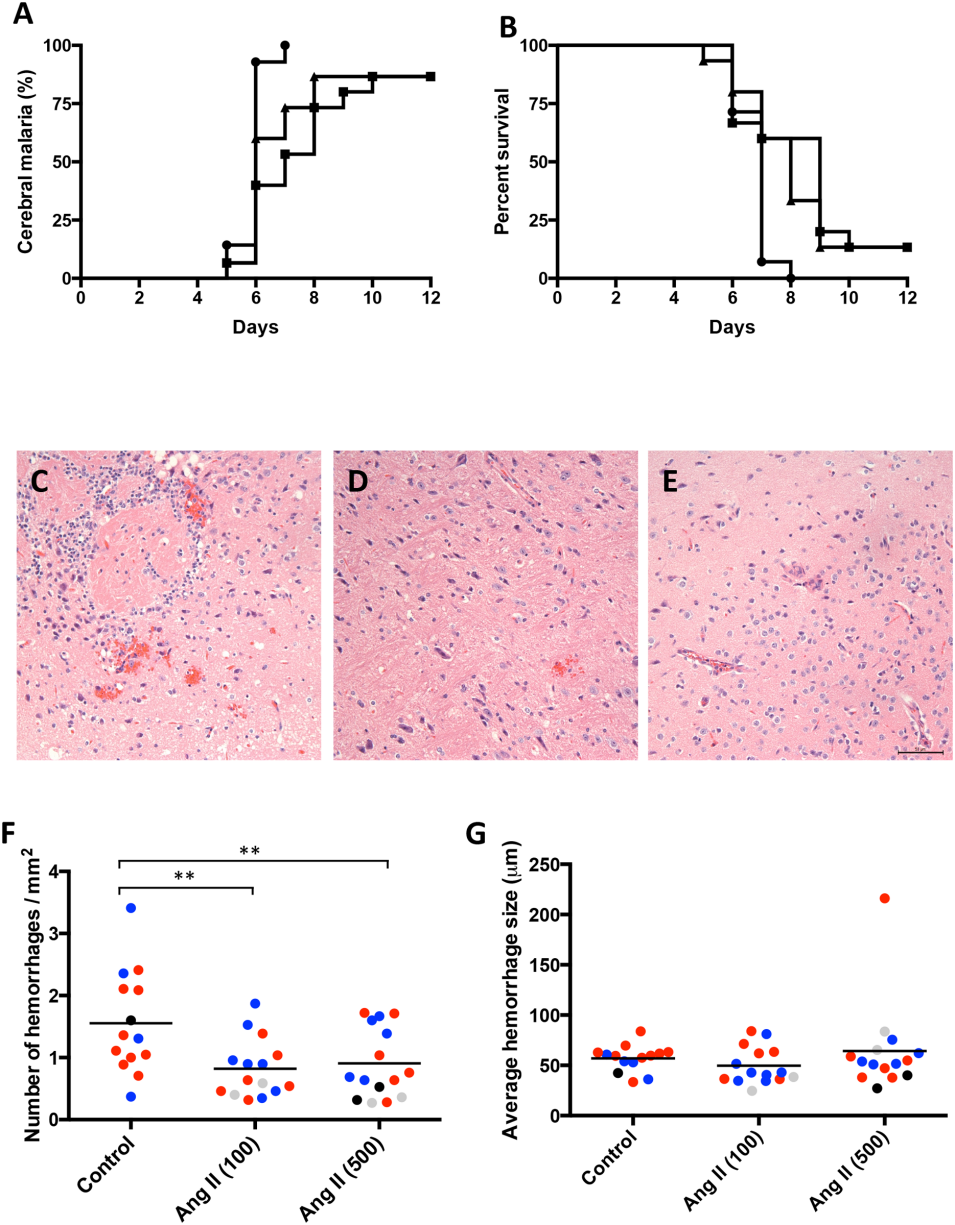
Ang II decreases brain hemorrhages, delays experimental cerebral malaria and increases survival of mice. Groups of mice as described in Fig 1C, control (black circles); Ang II at 100 and 500 ng/kg/min (black squares and triangles, respectively) were observed twice a day for determination of cerebral malaria based on neurological symptoms (as described in methods) (A) and survival (time of euthanasia) (B). Kaplan-Meier analyses were conducted to determine differences among the groups. Cerebral malaria and survival distribution of the groups were significantly different (*p*<0.010 and *p*<0.013, respectively). Pairwise log rank comparison after applying Bonferroni correction (statistical significance accepted at the *p*<0.0167 level) showed that for cerebral malaria there was a statistically significant difference between control *vs* Ang II (100) (*p* = 0.0021) but not *vs* Ang II (500) (*p* = 0.0258). For survival, both Ang II groups showed significant differences vs control group (*p* = 0.0087 and *p* = 0.0100 for Ang II (100) and Ang II (500) *vs* control, respectively). No significant differences were found between angiotensin groups, Ang II (100) and Ang II (500) (*p* = 0.511 and *p* = 0.524 for cerebral malaria and survival respectively). Histological sections of the brains of mice control (C), Ang II at 100 ng/kg/min (D) and Ang II at 500 ng/kg/min (E) were analyzed for the presence and size of hemorrhages. Bar is 50μm. Quantification of the number (F) and size (G) of hemorrhages per mm^2^. Mice that were sacrificed with a cerebral malaria score of 3 (black circles), 4 (blue circles) or 5 (red circles) and mice that did not develop cerebral malaria (gray circles) are indicated. Average values for each mouse are shown. **p<0.01 by ANOVA. No significant differences were observed between groups in (G).

Ang II moderately reduced the development of experimental cerebral malaria and increased survival. There was also a delay in the onset of symptoms of 1 to 2 days and a corresponding increase of survival time of these mice (Fig 2A and 2B).

Hemorrhages in the brain of each mouse were quantified in histological sections (Fig 2C–2E). Mice with elevated Ang II presented significantly lower densities of hemorrhages although of similar size when compared to control mice (Fig 2F and 2G). Mice that did not develop cerebral malaria in Ang II-treated groups show low numbers of hemorrhages, as expected because of the lack of neurological symptoms (Fig 2F).

It is possible that the Ang II-induced decrease in parasitemia contributed to the delay on the onset of experimental cerebral malaria, since the development of this syndrome is dependent on the levels of parasitemia [16]. Using the rodent malaria model of *P*. *berghei* infection, other authors observed that a treatment that inhibits Ang II generation (captopril, inhibitor of ACE) or one that inhibits one of Ang II receptors (losartan, antagonist for Ang II receptor type 1, AT1) resulted in decreased T cell responses and partial protection from cerebral malaria [17]. However, since no effects in the levels of parasitemia were observed after the losartan treatment, and only minor effects after captopril, it appears that the protective effects of these treatments may not be caused by direct inhibition of parasite growth. It is therefore possible that Ang II may have several independent effects on *P*. *berghei* infection, on one side inhibiting parasite growth as observed *in vitro* in this study and others [8, 11], on the another side facilitating protective immune or other mechanisms that affect the pathogenesis of experimental cerebral malaria [17], or even by acting on cardiovascular function by restoring blood pressure which is decreased in mouse [18] and human cerebral malaria [19–21]. The fact that Ang II presented a rapid effect on *P*. *falciparum* growth *in vitro*, while the effect in mice parasitemia is only observed after day 5 of infection may indicate differences in the Ang II effect on the two Plasmodium species and/or possibly that the growth-inhibitory effect of Ang II is not relevant *in vivo* and the late decrease of *P*. *berghei* parasitemia is due to indirect effects of Ang II on the immune response rather than direct killing of the parasite.

A protective role for Ang II against human cerebral malaria has been proposed based on a gene polymorphism analysis of angiotensin-related enzymes in patients with severe or mild malaria. It was observed that an insertion/deletion polymorphism of ACE, that is associated with increased levels of Ang II, was also strongly associated with mild malaria. Similarly, a mutation on angiotensin converting enzyme 2, that catalizes the conversion of Ang I or Ang II into Ang 1–7 and may result in increased Ang II in women, was also associated with mild malaria [7]. It is therefore possible that humans, as shown here with mice, also benefit from high levels of Ang II to decrease malaria pathogenesis.

## Conclusions

It was observed that elevated levels of Ang II have a beneficial effect against malaria-induced pathology in mouse models. Elevated Ang II moderately decreased the levels of parasite and the pathogenesis of the disease, resulting in lower number of hemorrhages and increased survival rates. These results are complementary to the previous observations in the human genetic study [7], suggesting that elevated levels of Ang II may be protective against malaria pathogenesis.

## Supporting Information

S1 Table Data and statistical analysis for all figures. (XLSX)

## Abbreviations

Ang II: Angiotensin II
ACE: Angiotensin converting enzyme
